# Acquisition System Based on Multisensors for Preserving Traditional Korean Painting

**DOI:** 10.3390/s19194292

**Published:** 2019-10-03

**Authors:** Taewon Choi, Soonchul Jung, Yoon-Seok Choi, Hyeong-Ju Jeon, Jin Seo Kim

**Affiliations:** Electronics and Telecommunications Research Institute, Daejeon 34129, Korea; taewon.choi@etri.re.kr (T.C.); s.jung@etri.re.kr (S.J.); ys-choi@etri.re.kr (Y.-S.C.); jeonhj@etri.re.kr (H.-J.J.)

**Keywords:** intangible cultural heritage, traditional painting, multisensor system

## Abstract

Rapid industrialization has significantly influenced people’s lifestyles in the recent decades, and the influence of traditional culture is diminishing. Recently, several studies attempted to simultaneously utilize various sensors to record delicate and sophisticated performances of intangible cultural heritage (ICH). Although painting is one of the most common ICH of human history, few research studies have recorded traditional painting work. In this paper, we aim to lay the groundwork for reviving Korean painting, even if there would be no painters to produce these traditional Korean paintings in the future. We propose a novel multisensor-based acquisition system that records traditional Korean painting work while minimizing interference in the work. The proposed system captures real-time data originating from the painter, brushes, pigments, and canvas, which are the essential components of the painting work. We utilized the proposed system to capture the painting work by two experts, and we visualize the captured data. We showed the various results of statistical analysis, and also discussed the usability.

## 1. Introduction

Intangible cultural heritage (ICH) includes traditions or living expressions that are inherited from our ancestors and passed on to our descendants; for example, oral traditions, performing arts, and skills required to make traditional crafts. Further, ICH is not a cultural manifestation, but rather the wealth of knowledge and skills passed on from one generation to the next. In recent decades, the lifestyle of people has changed drastically, and the influence of traditional culture on everyday life is diminishing [1]. Now, we can find many traditional cultures and products mostly in documentary films or in museums.

Many governments and organizations are attempting to preserve their ICH, which is in crisis. In particular, UNESCO listed out the ICH of the world and operated multimedia archives, eServices, to record these ICH [2]. However, such methods only record the external appearance of the performance of an expert, and higher-level data are required to revive the missing heritage information without any help from other experts.

Meanwhile, multisensor-based systems have been employed in various fields. Lei et al. introduced a fault detection method based on multisensor data fusion [3]. Dong et al. presented an overview of recent advances in multisensor satellite image fusion [4]. Yuan et al. designed a wearable multisensor system to obtain high-accuracy indoor heading estimations, according to a quaternion-based unscented Kalman filter algorithm [5]. Han et al. introduced a comprehensive approach for context-aware applications that utilize the multimodal sensors in smartphones [6]. Choi et al. proposed a multimodal sensor-based method for evaluating fear based on nonintrusive measurements [7].

Recently, novel approaches based on multisensors have been suggested to rigorously preserve ICH. In the European Union, experts in various fields including computer science, education, medical science, and physiology, from 12 organizations in seven countries, conducted the “i-Treasures Project”. They captured multimodal data such as dance motions, craft motions, brainwaves, and facial expressions in traditional singing, dance, craft, and composition. Further, they designed educational game-like applications for practicing different types of ICH expressions, based on the data [8,9]. Grammalidis et al. introduced the dataset created by conducting an i-Treasures project, including traditional dance moves captured with multiple Kinects or optical markers, and human beatboxes captured with a hyper-helmet and audio equipment. Magnenat-Thalmann et al. [10] also digitized folk dances originating from several regions of Europe, using an optical motion capture system as a recording device, and provided a learning framework for folk dances. Protopapadakis et al. [11] used Kinect to capture depth images and videos of six traditional Greek dances in order to identify key movements and gestures. Lombardo et al. presented a solution for drama preservation in terms of a formal encoding through computational ontology [12]. In addition, some researchers measured the learning effect of learners using a haptic device by capturing the process of manufacturing traditional paper [13].

**Figure 1:**
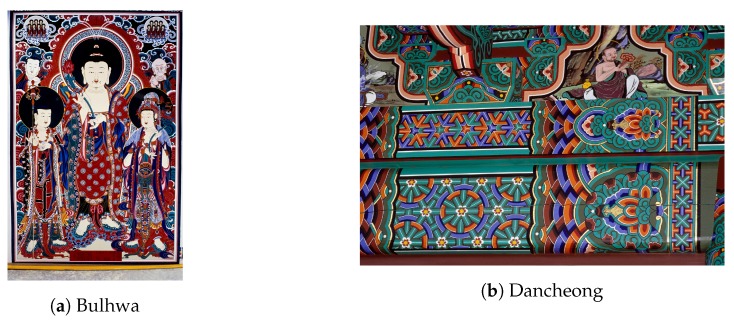
Two important branches in traditional Korean painting. Bulhwa means Buddhist painting which depicts Buddhas, bodhisattvas, and other relevant entities. Dancheong is traditional decorative coloring on wooden buildings.

Painting is one of the essential heritages for humankind; the history of painting dates back to prehistoric times and spans most cultures. This is also true for traditional Korean painting (Figure 1). Incidentally, the number of the successors has been decreasing on economic grounds, and we are experiencing difficulties in its transmission. However, there are few studies on capturing painting work to aid its transmission.

In this paper, we propose a novel multisensor-based acquisition system that records traditional Korean painting work with minimal interference. Painting work comprises the interactions between the following essential components; painter, canvas, brush, and pigment. They produce large amounts of different types of information, such as the painter’s pose, hand gesture, grasping power, brush pose, stroke shape, canvas material, and pigment concentration. Among them, we selected the painter’s action, canvas image, brush pose, and pigment information as the essential information for reviving traditional Korean painting. Thus, the proposed system captures this information.

Completing a traditional Korean painting requires a large amount of time, which implies that the proposed system should be sufficiently robust to unexpected external impacts. There have also been studies that acquired the data of brush motion by using a haptic device or attaching wired sensors to a brush [14,15]. Their objective was data acquisition for brush modeling, and they did not attempt to prevent interference with the painter’s work during acquisition. In contrast, we have utilized contactless sensors to avoid disturbing the painter’s work. As an exception, we had to attach a small marker tool, rather than a relatively heavy sensor, to a brush, because it was difficult to track the pose of the brush without any tools attached.

Further, we have encountered various difficulties in the process of building the proposed system. One of them is to configure the initial pose of a brush well. We have attempted to hold a brush upright by hand for the initial pose set-up, because canvases, in general, are laid out flat on a floor in traditional Korean painting, but there would always be an error of a few millimeters. Another problem is the time mismatch in the data captured from the sensors of the proposed system. Even if we started the sensors simultaneously, the recording time for the same event would differ from sensor to sensor. To analyze multimodal data properly, the sensors must be time-synchronized.

We utilized the proposed system to capture the painting work performed by two experts, and we visualize the captured data. Further, we show the results of statistical analysis, such as the total working time, drawing time, and the number of strokes.

The main contributions of this paper are as follows.

- We propose a new robust system for recording painting work using contactless sensors, which has not been attempted so far.
- We address the issue of the initial pose setup of a brush, which arises from the difficulty in holding the brush accurately upright.
- We address the time synchronization problem between two heterogeneous sensors.
- As a result, we lay the groundwork for preserving traditional Korean painting, which can easily be applied to other intangible cultural heritage related to painting.

The rest of this paper is organized as follows. We describe our proposed system in Section 2. We show and discuss the experimental results from the painting work by two experts in Section 3. Finally, we provide some concluding remarks in Section 4.

## 2. Proposed System

### 2.1. System Pipeline

**Figure 2:**
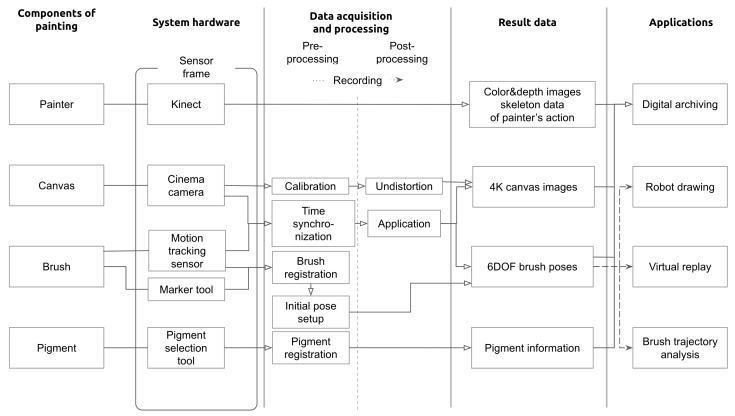
The pipeline of the proposed system. We consider painter, canvas, brush, and pigment as the essential components in painting. The proposed system is equipped with several sensors and tools to capture the important information that originated from them. Through preprocessing, recording, and postprocessing, the final result data is acquired. The result data has been utilized in various applications such as digital archiving, robot drawing, etc.

In this section, we describe how to build our system, preprocess before data recording, postprocess captured data, and apply the data. Figure 2 depicts the entire process of the proposed system.

As described previously, we decided to capture the painter’s action, canvas image, brush pose, and pigment information with a Kinect, cinema camera, motion-tracking sensor, and pigment selection tool, respectively.

**Figure 3:**
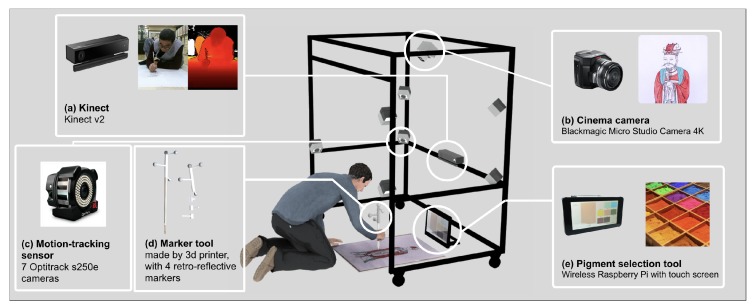
Hardware setup. The main structure of the proposed system is a cuboid-shaped aluminum frame equipped with contactless sensors such as a Kinect, a cinema camera, and a motion-tracking sensor. The marker tool attached to a brush is used by the motion-tracking sensor to detect the current pose of the brush. The pigment selection tool is used by the painter to specify the current pigment during painting.

We constructed a sensor frame to mount these sensors. As shown in Figure 3, the frame was designed to be cuboid-shaped and robust to external impacts, to prevent the mounted sensors from wavering back and forth while capturing the data. We describe the details of the frame in the following section.

We capture the painter’s skeleton and the color and depth images using Kinect. Because Kinect version 2 can record 1920 × 1080 color images and 512 × 424 depth images at 30 fps, it is fit for capturing the motions of the painter. We placed the Kinect in front of the painter, as shown in Figure 3a. The images captured by the Kinect are hardware-encoded through an NVIDIA 1080 Ti graphics processing unit and stored on a solid-state disk in real-time.

We use a cinema camera to match the canvas image, as shown in Figure 2. Because it should capture sophisticated pictures while covering a wide range, we require a high-resolution camera. Thus, we employ a Blackmagic Micro Studio Camera 4K, which can record video up to 3840 × 2160 resolution at 30 fps. We placed the cinema camera on top of the sensor frame such that it could record the canvas image better, as shown in Figure 3b. The video captured from the camera is hardware-encoded through an Intel HD graphics processing unit and stored on a solid-state disk in real-time.

Brush poses are captured by the motion-tracking sensor, which comprises seven OptiTrack infrared cameras. OptiTrack is an optical infrared marker-based motion-tracking sensor. Retroreflective markers reflect infrared light onto the infrared cameras. The system combines the captured data and calculates the 3D positions of all markers. By using a combination of several markers in a specific pattern, the system can identify rigid bodies that refer to objects that will not deform. The motion-tracking sensor identifies and tracks the brushes based on the rigid bodies at 120 Hz. We spread out the cameras on the frame to ensure that they could observe brushes clearly, as shown in Figure 3.

For tracking brushes, we constructed marker tools using a 3D printer. We mount each marker tool with four markers on each brush and register the markers as a rigid body, as shown in Figure 3d. The brush is very light, so attaching the marker tool could be a burden to the painter. To alleviate this burden, we make the marker tool as light as possible.

It is challenging to automatically identify the pigment applied by the painter on the canvas. Thus, the proposed system provides a wireless pigment selection tool, as shown in Figure 3e.

We describe the creation of the marker tool and the pigment selection tool in detail in the following subsections.

The process of acquiring a painter’s work data comprises three stages—preprocessing, recording, and postprocessing, as shown in Figure 2. Preprocessing includes calibration of the cinema camera, time synchronization between the cinema camera and the motion-tracking sensor, brush registration, initial pose setup of the brush, and pigment registration. Then, the system records the painting work. Finally, we postprocess the resulting canvas video and brush poses data.

In the preprocessing stage, we gather the necessary data before starting calibration and time synchronization. Subsequently, we calculate the calibrated camera matrix of the cinema camera for image undistortion. We determine the result of time synchronization between the motion-tracking sensor and the cinema camera. We must register the physical information of a brush before tracking it. We provide the details of the brush registration in the following subsection.

Lastly, we register the physical information of the pigments that will be used in the painting work in advance. This information includes the pigment’s name, photo, particle size, color, and raw material. This helps reproduce a captured painting work in other environments such as virtual reality applications.

**Figure 4:**
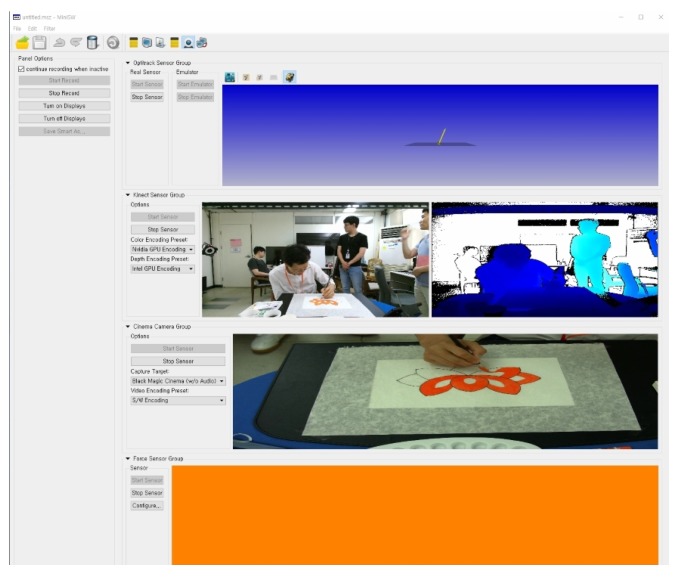
The program screen during the capture. The views show brush poses, color, and depth images from the Kinect; canvas images; and pigment information, respectively.

In the recording stage, the painter performs the work and the system records it. Figure 4 shows the program screen during the recording. The captured data are temporarily stored in the memory because, except for the video files, they are not very large. When the painter finishes the work, an operator ends the recording, and the data in the memory is stored on the hard disk.

The purpose of postprocessing is to apply calibration and time synchronization results to the captured data. We remove distortions in the video and synchronize the canvas video and the brush pose data.

Finally, as shown in Figure 2, we obtain color and depth video and skeleton data of the painter’s actions from the Kinect. We obtain 4K canvas images from the cinema camera, which are undistorted in postprocessing. We store the brush poses recorded by the motion-tracking sensor. Pigment information selected by the painter is stored with other data simultaneously.

Our main objective is to archive the acquired data to preserve the process of traditional Korean painting. Furthermore, we can utilize this data in several areas. For example, we can analyze brush trajectory data and extract meaningful information. We can utilize a robot to draw the brush trajectory for verification, and create entertainment applications in a virtual environment.

### 2.2. Hardware Configuration

#### 2.2.1. Sensor Frame

**Figure 5:**
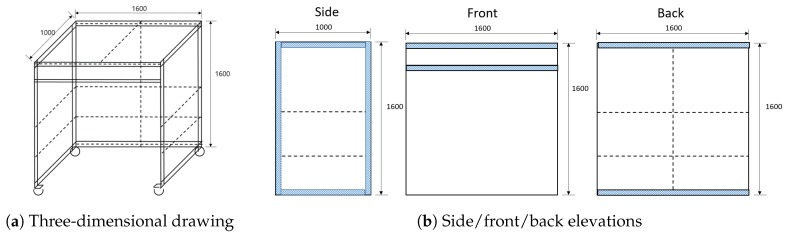
Sensor frame design. The frame is a 1.6 m (w) × 1. 6 m (h) × 1.0 m (d) sized cuboid-shaped aluminum frame equipped with large wheels, which has been designed to sufficiently be robust against internal and external shocks.

Figure 5 shows the design drawing of our sensor frame. We designed the sensor frame considering the following several points.

First, when determining the size of the sensor frame, we considered the canvas size and the minimum distance to prevent malfunctions of the motion-tracking sensor. The paintings in traditional Korean painting vary in size, and many of them are large enough to reach tens of meters. However, a one-meter canvas is sufficient for painters to fully show their painting skills. By adjusting the parameters of the motion capture sensor, we can reduce the operating distance to 40 cm. We can finally set the size of the sensor frame to 1.6 m (w) × 1.6 m (h) × 1.0 m (d).

Second, we attempt to design a robust frame, so that it does not move because of internal and external impact. Because the painting work usually takes more than tens of hours, the proposed system should be able to achieve stable operation for a long time. However, unexpected collisions with the staff or the painter are inevitable, and therefore we used profiles with 4 cm × 8 cm thickness for some parts that are less vulnerable to impact than the others where a 4 cm × 4 cm thickness was used. The combination of the profiles allowed to deal with impacts effectively; however, it is still not too thick and heavy to move quickly.

**Figure 6:**
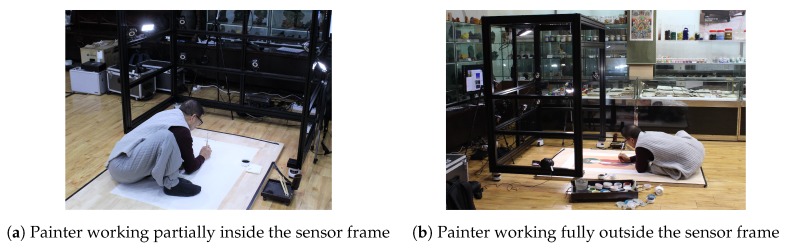
Typical painting work in the sensor frame.The sensors can capture the painting components, such as the painter, brush, and canvas, even outside the sensor frame. Therefore, the painter can work more freely.

Third, we consider the comfort of a painter when drawing a picture; therefore, one side of the cuboid-shaped frame is left open so that the painter can easily move around. As you can see in Figure 6, even if the painter does not enter the sensor frame entirely or works outside, the proposed system can capture his work. This feature allows the painter not to suffer from claustrophobia when he works for a long time and enables him to draw a picture that is much larger than the sensor frame. On the other hand, the disadvantage of the design is that it is vulnerable to the impact on the open side. To resolve this issue, we added a crossing bar on the top.

Fourth, we designed a frame to provide a so-called on-demand service. Artists usually prefer to work in their studios with various materials and tools required for the painting work. In traditional Korean paintings, artists use unique pigments that is challenging to handle (e.g., stone powder or gold powder). Besides, some painters are reluctant to show their skills in public places. Therefore, we decided to install the proposed system and capture their painting work in their studio. As the studio conditions are different from each other (e.g., room size, entrance size, and lighting condition), the frame is standardized for easy assembling and disassembling. Also, we equipped it with large wheels with leveling legs that can cause the frame to be adjusted to the ground for stability and mobility.

#### 2.2.2. Marker Tool

**Figure 7:**
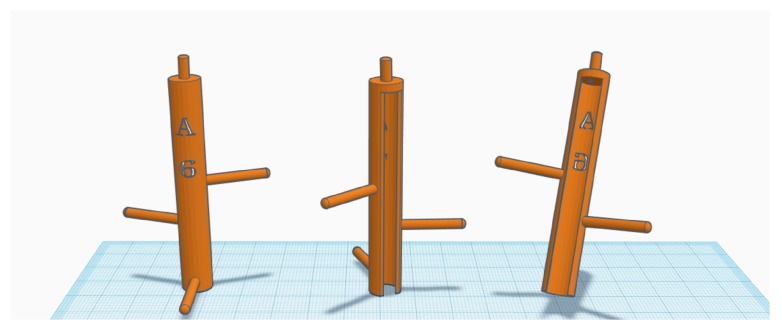
Marker tool design. The marker tools have been designed to be as light as possible, easily attachable/detachable, and easily observable.

As mentioned before, we attach a marker tool with four retroreflective markers to a brush and register the markers of the tool as a rigid body, so that the motion-tracking sensor can track the brush. Figure 7 shows the final design drawing of the marker tool. We designed the marker tool, considering the following several points.

**Figure 8:**
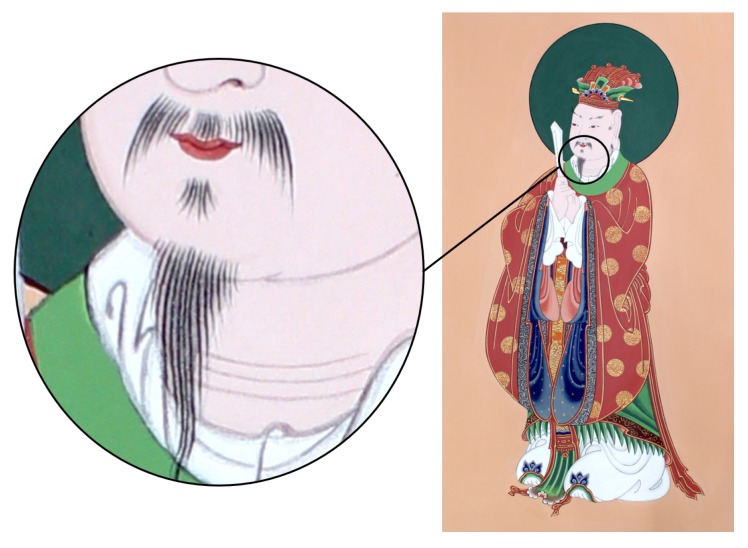
Detailed depiction of a Buddhist painting. The painters in traditional Korean painting require several tens of hours to create such a detailed depiction. This is why the marker tools should be as light as possible.

First, we designed the marker tool to be as light as possible. As mentioned previously, brushes are very light, and therefore attaching the marker tool can be a burden on the painter. As shown in Figure 8, the painters in traditional Korean painting must perform a very detailed depiction for a long time. We weighed brushes used by an expert, and we found that they are distributed between 3 g and 20 g. As the small full sphere marker attached to a marker tool weighs ~1 g, the marker tool may be heavier than small brushes. We printed the marker tools with polylactic acid (PLA) material, and to make them as light as possible, we hollowed out the inside and reduced the thickness. As a result, we were able to produce a marker tool weighing ~2 g, excluding the markers.

Second, the marker tool is designed so that it can be easily attached to and detached from a brush. After finishing the painting work, painters often reuse their brushes for other works. Therefore, we do not permanently attach a marker tool to a brush; we designed the marker tool shaped like a semicircle, as shown in Figure 7. In practice, the marker tool can be firmly attached to a brush by one touch, and it is easily removable.

Third, we designed a marker tool such that the motion-tracking sensor could easily observe the attached markers. Because the brush handle could occlude the markers from the sensor if they were located too close to the brush, we created a type of arms on the marker tool, and then, the markers could be placed slightly away from the brush. This feature helps the motion-tracking sensor to observe the markers better.

**Figure 9:**
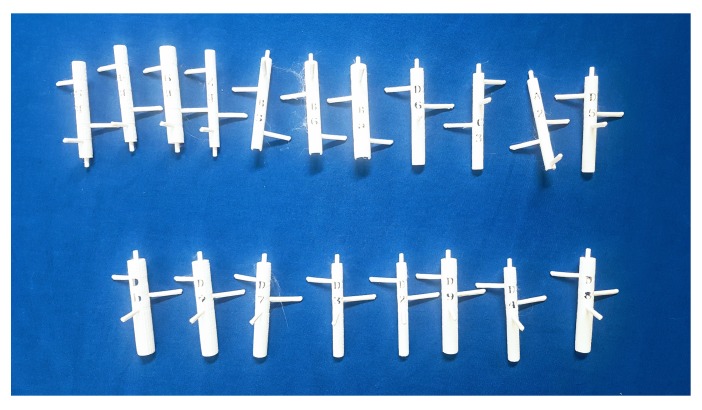
Marker tools. We used an inexpensive 3D printer to create marker tools with various sizes and patterns.

**Figure 10:**
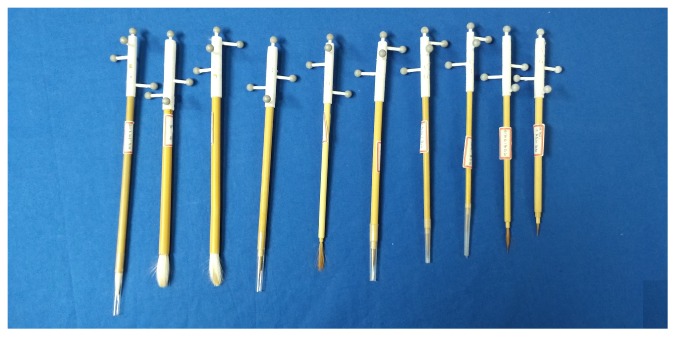
Brushes assembled with marker tools.

Fourth, we made marker tools in various sizes to fit different brushes instantly. In general, painters use many brushes in their work, and they may need to attach a marker tool to an unknown brush. Therefore, it is necessary to prepare many marker tools of various sizes. Figure 9 shows marker tools with the same pattern but with different diameters. Figure 10 shows the brushes with a marker tool attached.

Finally, the top marker of a marker tool was designed to be positioned on a straight line passing through the brush handle. This design helps resolve the issue of the initial pose setup of brushes.

#### 2.2.3. Pigment Selection Tool

**Figure 11:**
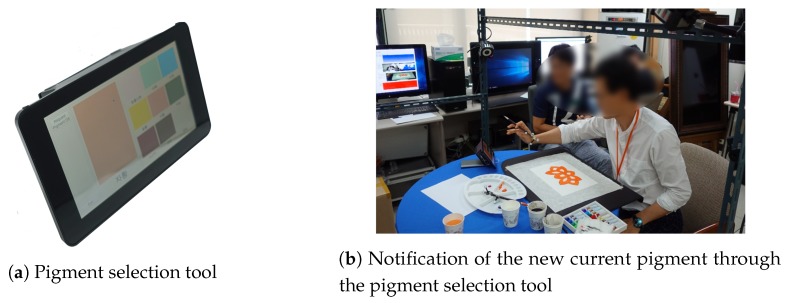
Pigment selection tool and its working example. The pigment selection tool is a Raspberry Pi equipped with a touch screen and a battery. It communicates with the PC through Wi-Fi, which removes the restrictions on the location of the tool.

It is difficult to automatically identify the pigment with which the painter is daubing the canvas using computer-vision techniques. Further, these techniques may fail to identify the pigment in some scenarios. Therefore, the proposed system provides a wireless pigment selection tool. The pigment selection tool is comprised of a Raspberry Pi equipped with a touch screen and a battery, as shown in Figure 11a.

Before using this tool, we register the pigment information in the proposed system. The registered pigment information is displayed on the touch screen. When the painter daubs the canvas with a new pigment, the information about the new pigment can be notified to the system by touching the corresponding icon in the pigment selection tool. Figure 11b shows the painter notifying the new pigment using the pigment selection tool.

### 2.3. Data Processing

#### 2.3.1. Brush Registration

For the motion-tracking sensor to track pose of the brush with six degrees of freedom (DOF), it is necessary to register the four markers on the brush as a rigid body. The pose of the brush comprises the position and orientation of the brush. A pivot point of the rigid body is a spot where the rigid body is rotated.

We define the position of the brush tip as the pivot point of the rigid body during the registration. Note that when the painter draws a picture with the brush, the position of the pivot point of the brush captured by the motion-tracking sensor is slightly different from the real position of the brush tip. This is because the hair of the brush is not a rigid body and bends quickly. Nevertheless, the pivot point is meaningful, and we can derive the approximate trajectory of the brush using this point. To do so, we temporarily attached a small marker at the tip of the brush. Note that once we set a pivot point, we do not maintain the attached marker any longer.

We also store the additional information such as its name, handle length, photo, type, length of the brush hair, and the thickness of the brush hair.

#### 2.3.2. Initial Pose Setup of a Brush

Before the acquired data is utilized for general applications, it is necessary to set the initial pose of the brush. The initial pose here means the physical pose of an object without any rotation and translation. If the initial poses are different from each other, there may be unexpected results when using the acquired data. For example, suppose that we set the initial pose of the brush vertically while it is set as horizontal in the robot painting application. In this case, if we rotated the brush about the x-axis by 90 degrees, it would stand horizontal. On the other hand, the robot painting application would lead to the brush standing vertical in the reverse direction for the same event.

In general, the canvas is laid out flat on the floor in traditional Korean painting. We regarded the upright pose of a brush as its initial pose. The issue at this point is that it is challenging to set the brush accurately upright without expensive equipment. When we attempt to hold the brush upright with our hand, there is always be an error of a few millimeters. Further, it is not easy to correct the error by moving the brush by hand.

**Figure 12:**
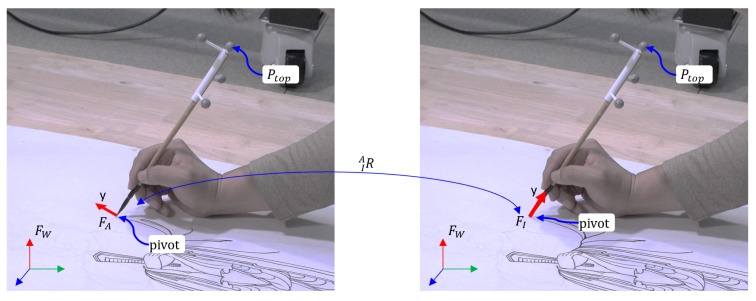
Relationship among the coordinate frames during painting. $F_{W}$ is the world coordinate system. $F_{A}$ is the model coordinate frame created by setting the initial pose of the brush when it is in an arbitrary pose. $F_{I}$ is the ideal model coordinate frame created by setting the initial pose of the brush when it stands upright; therefore, the y-axis of $F_{I}$ always passes through the brush handle.

Therefore, we propose a new method to obtain a consistent result without physically holding a brush upright. Our idea is to derive the transformation between the arbitrary initial pose configured from the motion-tracking sensor and the ideal initial pose by measuring the orientation of the brush. Let us start with a simple example. Figure 12 shows two identical scenes while a painter is painting. $F_{W}$, here, is the world coordinate system. The motion-tracking sensor reports the poses of rigid bodies and the positions of markers relative to $F_{W}$. $F_{A}$ is the model coordinate frame created by setting the initial pose of the brush when it is in an arbitrary pose. Therefore, we can not derive any explicit relationship between $F_{A}$ and the brush, as shown in the left figure. On the other hand, $F_{I}$ is the ideal model coordinate frame created by setting the initial pose of the brush when it stands upright. However, it is almost impossible to set the brush accurately upright as described above. Further, as shown in the right figure, the y-axis of $F_{I}$ always passes through the brush handle. The motion-tracking sensor knows about $F_{A}$, but not $F_{I}$, which we want to know. By deriving ${}_{I}^{A}\;R$, which is the transformation between $F_{A}$ and $F_{I}$, we can acquire the poses of the brush whose initial pose is assumed to stand upright.

To derive the transformation, we specially designed the marker tool to position the marker at the top of the brush, so that the motion-tracking sensor would report the top position of the brush. Further, we temporarily attached a small marker to the tip of the brush. Therefore, we can obtain the orientation of the brush.

The relationship between $F_{W}$ and $F_{A}$ is

(1) $${}^{W}\;P={}_{A}^{W}\;R\cdot {}^{A}\;P+{}_{A}^{W}\;T$$

where ${}^{A}\;P$ and ${}^{W}\;P$ are the model coordinate and the world coordinate of point *P*, respectively, and $\left({}_{A}^{W}\;R,{}_{A}^{W}\;T\right)$ is the pose of $F_{A}$ relative to $F_{W}$. $P_{top}$ is the top point of the brush, and ${{}^{W}\;P}_{top}$ is the coordinate of $P_{top}$ with respect to $F_{W}$. Note that the motion-tracking sensor always reports the values of ${}_{A}^{W}\;R,{}_{A}^{W}\;T,$ and ${{}^{W}\;P}_{top}$. h is the distance from the pivot point (${}_{A}^{W}\;T$) to the top point (${{}^{W}\;P}_{top}$) of the brush and can be obtained from a single observation.

The relationship between $F_{W}$ and $F_{I}$ is expressed as

(2) $${}^{W}\;P={}_{I}^{W}\;R\cdot {}^{I}\;P+{}_{I}^{W}\;T$$

where ${}^{I}\;P$ is the coordinate of a point, *P*, and $\left({}_{I}^{W}\;R,{}_{I}^{W}\;T\right)$ is the pose of $F_{I}$ relative to $F_{W}$, which is what we attempt to achieve. From $P_{top}$ and the relationship between Equations (1) and (2), we can derive $\left({}_{I}^{W}\;R,{}_{I}^{W}\;T\right)$. Because the same tip end has been defined as the pivot point of the two model coordinate frames $F_{A}$ and $F_{I}$, it satisfies ${}_{A}^{W}\;T={}_{I}^{W}\;T$. Thus, for a point, *P*, the following equations can be derived from Equations (1) and (2),

(3) $$\begin{array}{ccc} {}_{A}^{W}\;R\cdot {}^{A}\;P+{}_{A}^{W}\;T & = & {}_{I}^{W}\;R\cdot {}^{I}\;P+{}_{I}^{W}\;T \end{array}$$

(4) $$\begin{array}{ccc} {}_{A}^{W}\;R\cdot {}^{A}\;P & = & {}_{I}^{W}\;R\cdot {}^{I}\;P \end{array}$$

(5) $$\begin{array}{ccc} {}_{A}^{W}\;R\cdot {}^{A}\;P & = & {}_{A}^{W}\;R\cdot {}_{I}^{A}\;R\cdot {}^{I}\;P \end{array}$$

(6) $$\begin{array}{ccc} {}^{A}\;P & = & {}_{I}^{A}\;R\cdot {}^{I}\;P \end{array}$$

This means that only if we derive the rotation matrix ${}_{I}^{A}\;R$, we can obtain ${}_{I}^{W}\;R$, which transforms points from $F_{I}$ into $F_{W}$.

**Figure 13:**
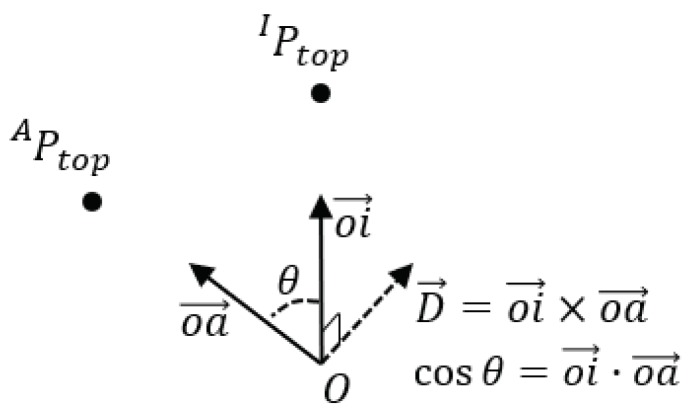
Components for deriving ${}_{I}^{A}\;R$. Because we know the position of the brush top in the coordinate frame $F_{A}$ and $F_{I}$, respectively, we can derive the rotation ${}_{I}^{A}\;R$, which transforms a point from $F_{I}$ into $F_{A}$.

Because brushes are rigid bodies, the transformation between frames $F_{A}$ and $F_{I}$ is also rigid. Therefore, ${}_{I}^{A}\;R$ is constant. We derive ${}_{I}^{A}\;R$ by computing the axis-angle representation $\left(\vec{D},\theta \right)$, which transforms the point of the brush top $P_{top}$, from $F_{I}$ into $F_{A}$. Figure 13 shows several components to derive ${}_{I}^{A}\;R$. We can see that ${{}^{A}\;P}_{top}={{}_{A}^{W}\;R}^{-1}\cdot \left({}^{W}\;P_{top}-{}_{A}^{W}\;T\right)$ from (1). Given $h=|{{}^{A}\;P}_{top}|$, the coordinate of the brush top relative to $F_{I}$ is, by definition, ${{}^{I}\;P}_{top}={\left(0,h,0\right)}^{T}$. $\vec{oa}$ is the unit vector from the origin to ${{}^{A}\;P}_{top}$, and $\vec{oi}$ is the unit vector from the origin to ${{}^{I}\;P}_{top}$. Here, $\left(\vec{D},\theta \right)$ is defined as

(7) $$\begin{array}{ccc} \vec{D} & = & \vec{oi}\times \vec{oa} \end{array}$$

(8) $$\begin{array}{ccc} \theta & = & \arccos\vec{oi}\cdot \vec{oa} \end{array}$$

From Equations (1)–(8), we can get the current pose of the brush, $\left({}_{I}^{W}\;R,{}_{I}^{W}\;T\right)$, whose initial pose is standing upright.

#### 2.3.3. Time Synchronization between the Cinema Camera and Motion-Tracking Sensor

When we acquire multimodal data from two or more sensors, in general, the record-starting times of the sensors are slightly different from one another. Such discrepancies may not be concerning if the data obtained from one sensor are utilized independently of the data collected from the other sensors. However, in our case, time synchronization is essential because the canvas videos and brush trajectories are often utilized simultaneously.

The time synchronization problem between sensors has been mainly studied in the case of sensors on a wireless network [16,17]; however, as the proposed system does not use a wireless network, it is not suitable for applying their method to the proposed system.

In this section, we show how to synchronize the canvas camera and the motion-tracking sensor. As data generated by the other sensors are also mostly video, they can be synchronized with the proposed method.

**Figure 14:**
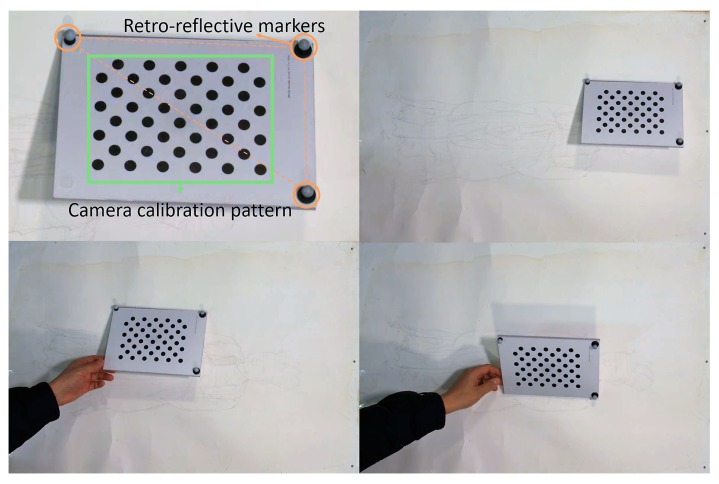
Preliminary data acquisition for the time synchronization between the motion-tracking sensor and the canvas camera. The pattern board contains three retroreflective markers detected by the motion-tracking sensor and a cycle pattern detected by the canvas camera. We repeatedly moved and stopped the pattern board to obtain the respective signals from the two sensors.

We use a pattern board with retroreflective markers for both camera calibration and synchronization, as shown in Figure 14. The motion-tracking sensor acquires the poses of the pattern board employing the markers, and the canvas camera records the pattern board. We analyze the recorded images and calculate the positions of the center point of the pattern in pixels. Note that the position of the pattern board obtained from the motion-tracking sensor is measured in millimeters.

Because both the markers and pattern belong to the pattern board, they always move at the same time; the board does not wander on the motion-tracking sensor, and neither does it wander on the canvas camera. When the board starts to move, its position also begins to change on both of the sensors.

As shown in Figure 14, we leave the pattern board still for a while, and then move it to another location. We repeated the action to obtain the data for synchronization. Empirically, recording for ~2 min is enough for analysis.

We obtain the optimal time difference by applying cross-correlation to the data from the two sensors. Cross-correlation is a method for measuring the similarity of two signals [18], and it defined as follows respect to the continuous signals *a* and *b*,

(9) $$\left(f*g\right)\left(\tau \right)=\int_{-\infty }^{\infty }f(t)g(t+\tau )dt$$

Here, τ is the displacement, also known as lag. *i* is always equal to or greater than zero and discrete. The Equation (9) can be transformed to the discrete form as

(10) $$\left(f*g\right)\left[\tau \right]=\sum_{i=0}^{n}f\left(i\right)g\left(i+\tau \right)$$

where *i* is greater than or equal to zero.

**Figure 15:**
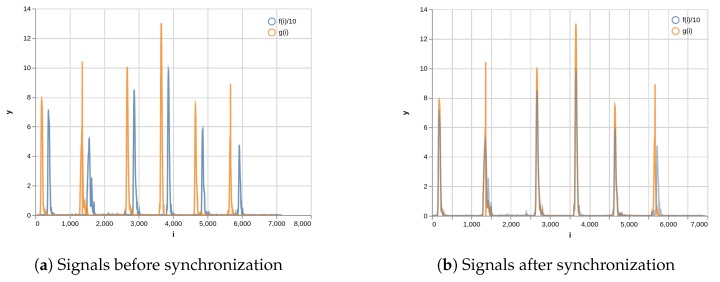
Signals before and after synchronization. The blue line means the function, *f(i)*, of the position changes of the pattern board monitored by the motion-tracking sensor; the red line means the function, *g(i)*, of the position changes of the pattern board detected by the canvas camera.

Before defining the signals f(t) and g(t), we first need to normalize the sampling rate of both data acquired from the two sensors. As mentioned above, as the canvas camera generates 30 fps videos, the positions of the pattern center point are sampled at 30 Hz. The motion-tracking sensor reports the poses of a rigid body composed of retroreflective markers at 120 Hz. We normalize the positions of the pattern center point at 120 Hz by linear interpolation. Under these conditions, the signals are defined as follows,

(11) $$\begin{array}{c} f\left(i\right)=∥\vec{p_{i}}-\vec{p_{i-1}}∥ \end{array}$$

(12) $$\begin{array}{c} g\left(i\right)=∥\vec{o_{i}}-\vec{o_{i-1}}∥ \end{array}$$

where $\vec{p_{t}}$ is *i*th pixel coordinate of the central pattern point acquired from the canvas camera of the pattern, and $\vec{o_{t}}$ is *i*th coordinate of the pattern board acquired from the motion-tracking sensor in millimeters. f(i) and g(i) denote the velocities of the pattern board at the *i*th step as observed from the two sensors, respectively. Figure 15a shows a graph plotting f(i)/10 and g(i). To make it easier to see, we scaled f(i) appropriately. Although the acquiring sensors are different from each other, the trends of the two functions should inevitably look similar because both functions represent the velocity of the pattern board with the markers. It is clear that there is a time lag between the two signals.

**Figure 16:**
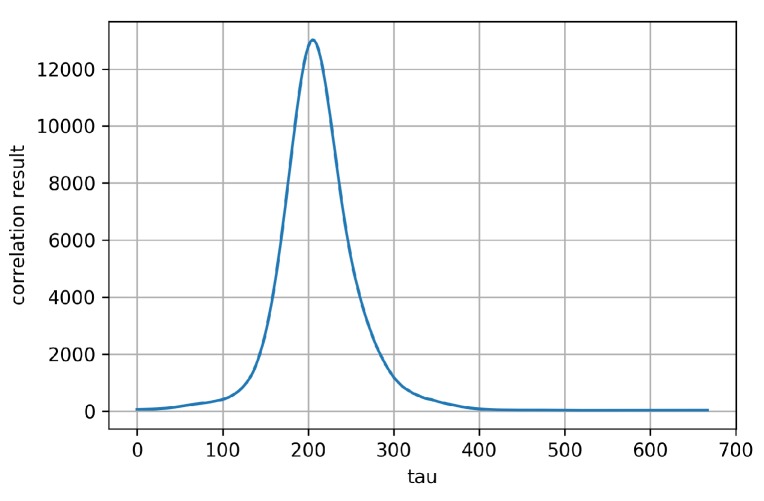
Result of cross-correlation. Cross-correlation measures the similarity of two signals. τ is the time lag, which is applied to g(i). We can see that the two signals have the highest similarity when τ=205.

Figure 16 is a graph of plotting the cross-correlation result of (f∗g)[τ]. It shows that the highest correlation is around τ=205. Figure 15b shows a graph plotting f(i)/10 and $g^{\prime }\left(i\right)=g\left(i+\tau \right)$ when τ=205. The figure indicates that the patterns of the two functions are very consistent, which means that these two data are now in sync. As the sampling rate of the data is 120 Hz, the time difference between the two original data is τ/120=1.71 s.

## 3. Experimental Results

**Figure 17:**
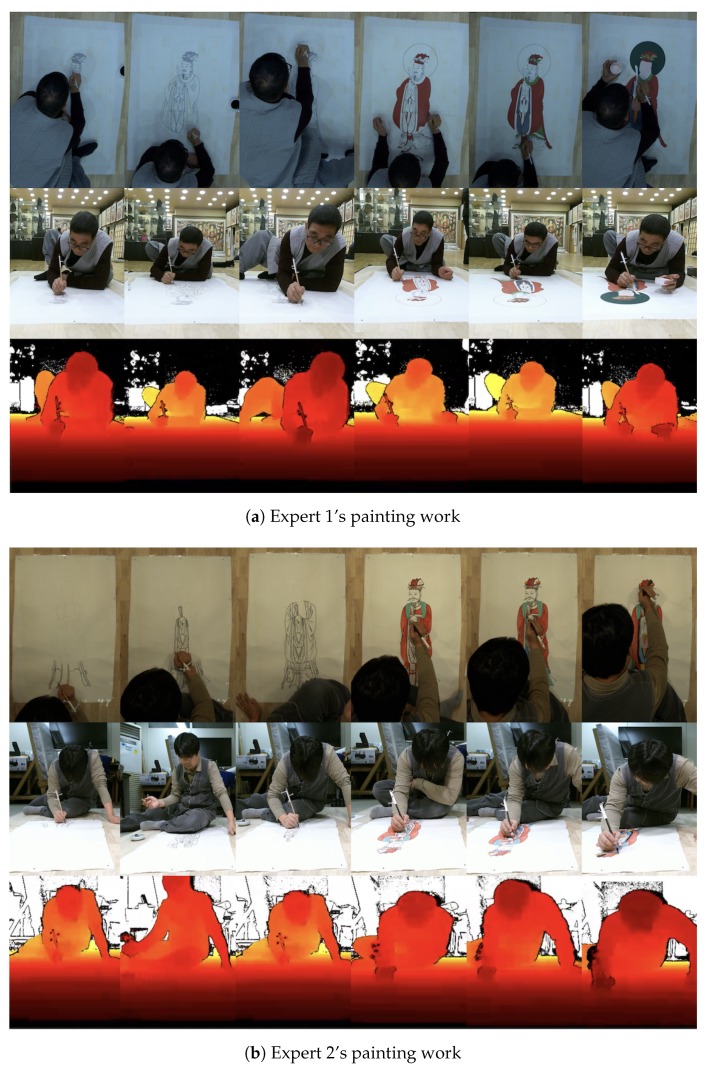
Two experts’ painting work. The top images are from the canvas cinema camera. The middle images are color ones from the Kinect. The bottom images are depth images from the Kinect.

We requested two professionals in traditional Korean painting to draw Siwang, one of the major gods of Buddhism. We captured their activities and tracked their brush stroke. Figure 17 shows the series of images captured in the painting work by Expert 1 and Expert 2. The top line shows the canvas images acquired from the canvas camera located at the top of the head. The middle and bottom lines show the color and depth images, respectively, captured by the Kinect sensor.

We divided the brush poses into two classes—strokes and others. Strokes are marks made by drawing a brush in one direction across the canvas. Recall that we set the tip of the brush as the pivot point. If a series of pivot points is observed under the canvas, those points compose a stroke. By doing so, we can extract useful information such as the number of strokes and the time interval for each stroke. Table 1 summarizes the experimental results. In this experiment, we analyzed both sketching and coloring, respectively. Cho is the phase of the sketch using a relatively thin brush and a black pigment. Chaesaek is the phase of applying colors and drawing patterns. The column *Number of strokes* denotes the number of strokes found during the two experts’ cho and chaesaek work of the two experts, respectively. The column *Stroke length* denotes the length of a stroke, which is calculated as

(13) $$Stroke\;length=\sum_{i=1}^{n-1}\left|\right|p_{i+1}-p_{i}\left|\right|$$

where $p_{i}$ is the coordinate of the *i*th point in the stroke. We derived several statistics, such as the mean, standard deviation, maximum, minimum, and sum from the stroke lengths.

The column “Stroke time” denotes the time it takes to draw each stroke. If we denote by $t_{i}$ the creation time of the *i*th point in a stroke, we can calculate stroke time as

(14) $$Stroke\;time=|t_{n}-t_{1}|$$

where $t_{1}$ and $t_{n}$ mean the start and end times, respectively, in the the stroke. Similarly, we determined the mean, standard deviation, maximum, minimum, and sum for stroke times. Moreover, we added the column “Work time total”, which includes all the time for drawing, preparing pigments, or waiting for the pigment to dry.

The column “Stroke speed” denotes the stroke drawing speed, which is the ratio of its total stroke length and total stroke time. We figured out the standard deviation and the maximum stroke speed.

Let us compare Expert 1 and Expert 2. The average stroke lengths of Expert 1 are 20.71 mm and are 7.51 mm longer than those of Expert 2 in the cho and chaesak phases, respectively. Expert 1 tends to draw longer strokes than Expert 2. The average stroke times of Expert 1 are 7.28 s; 5.17 s longer than those of Expert 2 in the cho and chaesak phases, respectively. Expert 1 spends more time to draw longer strokes than Expert 2. Expert 1 is slower than Expert 2 in terms of stroke speed. From the analysis of the results, we could distinguish the work styles of the two experts even though they did not draw the same picture.

Let us compare cho and chaesaek. In terms of stroke speed, both experts drew chaesaek faster than cho. In the work of Expert 2, the mean stroke length in the chaesaek phase is even almost three times longer than that in the cho phase. It implies that chaesaek is easier to draw than cho.

Using the data, we can obtain the ratio of the total work time to the actual drawing time. In addition to the actual drawing time, the total work time involves preparing pigments, applying pigment to the brush, and waiting for the canvas to dry. The ratios of chaesaek are larger than those of cho. For Expert 1, this ratio of cho is ~1.5; however, for chaesaek, it gets closer to 2. For Expert 2, this ratio of cho and chaesaek is approximately 2.1 and 4.9, respectively. The reason why this trend is stronger in chaesaek than in cho is that the experts use more pigments and brushes in this phase.

**Figure 18:**
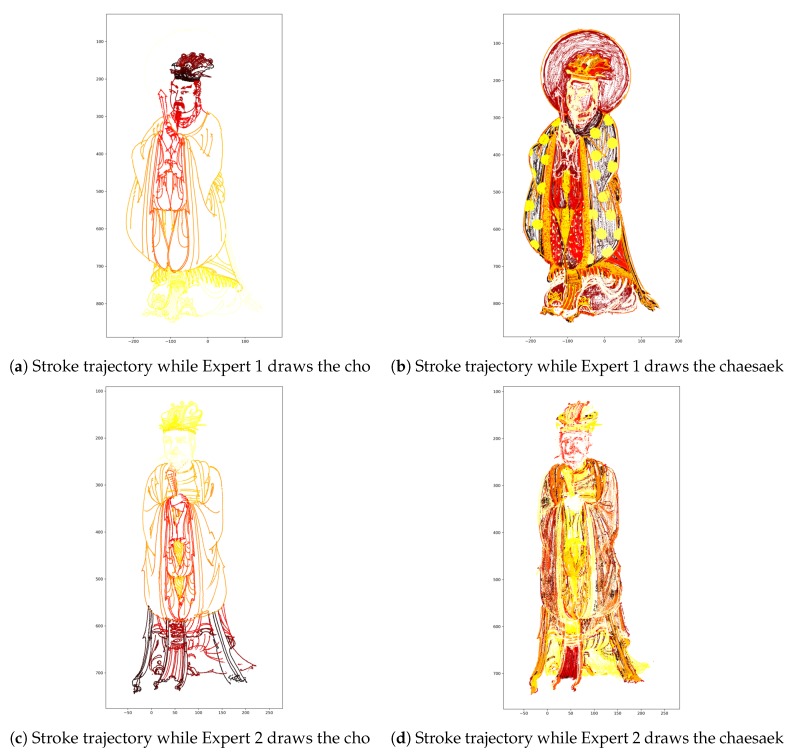
Visualization of stroke trajectories in each painting work, obtained by plotting the dots corresponding to the points of the strokes. The colors of the dots denote the progress of the drawing according to time. Black, red, and yellow indicate the early, middle, and final sections during painting, respectively.

Because we acquired time series data, we can identify the order in which the painter draws the picture. Figure 18 visualizes the painting sequence. We plot the x- and y-axes of the stroke trajectory. The series of data is filtered by the z-axis with a threshold of the height from the ground. This implies that we filtered the data; we omit the data recorded when the brush hair is on the canvas. Colors denote the progress of the drawing in the time axis: black, red, and yellow indicate the early, middle, and final sections during painting, respectively.

In the cho phase, Expert 1 went down from top to bottom while Expert 2 climbed from bottom to top. The case of chaesaek looks somewhat complicated. In fact, it consists of various small steps. In general, an expert covers primary colors throughout the canvas, shades some parts, and finally, adds some patterns in the chaesaek phase. However, it is not clear how to classify these steps and the work order of the steps varies from expert to expert. In the chaesaek phase of Expert 1, a yellow circular pattern stands out because he adds gold patterns in his final step. On the other hand, in Expert 2’s work, the end of the person’s clothing appears to be highlighted because he partly shaded it at the end of the work.

**Figure 19:**
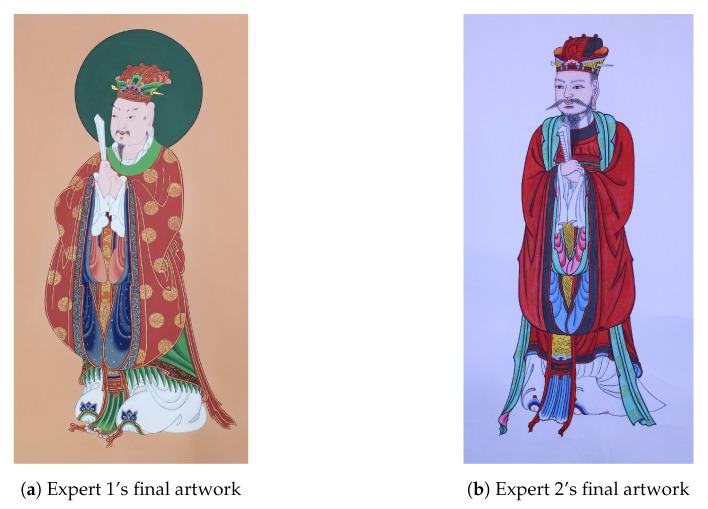
Two experts’ final work. We can see that the two experts are different from each other in style.

Figure 19 shows the resulting images of their work. Previously, we only can guess the process of painting work. However, we have now shown that we could analyze the details of the work progress with the proposed system.

**Figure 20:**
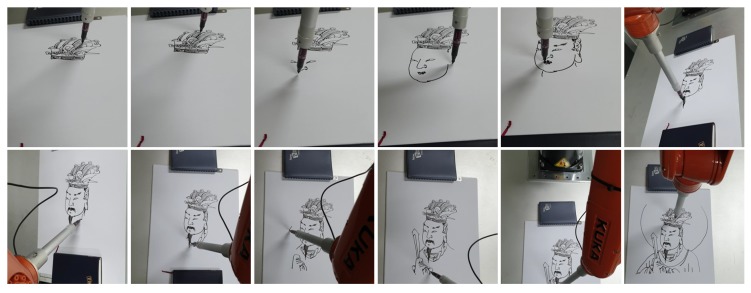
An example of applications utilizing the acquired data: Robot drawing. The robot imitates the expert’s brush movements.

**Figure 21:**
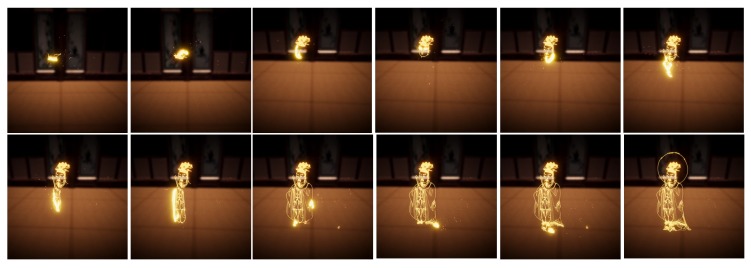
Another example of applications: Virtual replay.

Archiving the data acquired in this way is very meaningful. Even if traditional Korean paintings were not passed on in the future, the data could be used as a stepping stone to revive again. In addition, it can be widely used in various fields. Figure 20 shows a case where the robot redraws the drawing using Expert 1’s brush pose data obtained by the proposed system. Figure 21 shows the viewer replaying Siwang drawn by Expert 1 in a virtual reality environment. In this case, we can observe their work at a point where it was never possible before, and make the reproduction work a cultural commodity.

We also expect to be able to analyze and use the acquired data for a variety of problems, as we showed in this section. For example, the data can be used to judge the falsification of a drawing by analyzing the style and strokes of a painter. Besides, we expect to classify the proficiency of a painter by capturing and analyzing work of various levels of painters, such as how to control a brush consistently, how to keep the angle between canvas and brush constant.

As we have applied the proposed system to the field, we found some unexpected limitations. One example is the issue of the coloring technique called barim. Painters use several brushes during painting. However, they usually use only one brush in a hand. The proposed system determines the active brush by considering the speed and position of the brushes detected by the motion-tracking sensor. Barim is a technique for creating gradation effects by applying pigments and spreading them with water. At this time, painters hold two brushes in a hand and use a pigment on one brush and water on another brush. Then, they draw their drawings using the two brushes alternately. The proposed system did not consider this situation, and as a result, the experts had to abandon their habit and work uncomfortably.

## 4. Conclusions

In this paper, we proposed a novel multisensor-based acquisition system that captures the core work of traditional Korean painting while minimizing interference in an expert’s work. The proposed system records the information of four components of painting work—painter, canvas, brush, and pigment. To record the information, the proposed system consists of a sensor mounting frame, contactless sensors to capture data, a marker tool, and a pigment selection tool. We described how to solve the issues of the initial brush pose setting and time synchronization between the two sensors.

We utilized the proposed system to capture the painting work from two experts. We then visualize the acquired data, and we show some statistics. As a result of the investigation of the acquired data, it was found that there were both similar and different attributes between the two professional painters. We also discussed another usability of acquired data.

Our future works are as follows. We are attempting to solve new issues discovered during the acquisition of the two experts’ painting work. We plan to remove the painter’s body or hands to acquire clear canvas images by applying background estimation methods, such as the authors of [19]. We believe that capturing the information of moving hands, such as in [20], is important, so we will make efforts to derive the information by combining depth and canvas images.

## Figures and Tables

**Table 1:** Experimental results on strokes from two experts’ work. Cho and Chaesaek mean the sketching and coloring phases, respectively.

		Expert 1		Expert 2	
		Cho	Chaesaek	Cho	Chaesaek
Number of strokes		179	1494	395	868
Stroke length (mm)	Mean	30.90	37.82	10.19	30.31
	STD	34.32	49.59	12.29	48.64
	Max	222.87	500.15	74.21	350.08
	Min	0.66	0.28	0.30	0.15
	Total	5,531	56,496	4025	26,307
Stroke time (s)	Mean	11.08	10.18	3.80	5.01
	STD	8.00	9.57	3.29	6.35
	Max	45.99	94.40	18.86	45.24
	Min	0.30	0.04	0.05	0.04
	Total	1983.21	15,208.90	1501.23	4348.33
	Work time total	2982.00	30,920.00	3106.00	21,224.00
Stroke speed (mm/s)	Mean	2.80	4.95	3.15	7.18
	STD	2.14	8.44	2.68	8.18
	Max	12.65	186.69	27.56	91.11
